# Wide-field tissue polarimetry allows efficient localized mass spectrometry imaging of biological tissues†
†Electronic supplementary information (ESI) available. See DOI: 10.1039/c5sc03782d


**DOI:** 10.1039/c5sc03782d

**Published:** 2015-12-15

**Authors:** Alessandra Tata, Adam Gribble, Manuela Ventura, Milan Ganguly, Emma Bluemke, Howard J. Ginsberg, David A. Jaffray, Demian R. Ifa, Alex Vitkin, Arash Zarrine-Afsar

**Affiliations:** a Techna Institute for the Advancement of Technology for Health , University Health Network , Toronto , ON M5G-1P5 , Canada . Email: arash.zarrine.afsar@utoronto.ca; b Department of Medical Biophysics , University of Toronto , 101 College Street Suite 15-701 , Toronto , ON M5G 1L7 , Canada; c STTARR Innovation Centre , Princess Margaret Cancer Centre , 101 College Street , Toronto , ON M5G 1L7 , Canada; d Department of Surgery , University of Toronto , 149 College Street , Toronto , ON M5T-1P5 , Canada; e Keenan Research Centre for Biomedical Science , Li KaShing Knowledge Institute , St. Michael's Hospital , 30 Bond Street , Toronto , ON M5B-1W8 , Canada; f Department of Chemistry , York University , 4700 Keele Street , Toronto , ON M3J-1P3 , Canada; g Department of Radiation Oncology , University of Toronto , 610 University Avenue , Toronto , Ontario M5G 2M9 , Canada; h Division of Biophysics and Bioimaging , Ontario Cancer Institute , University Health Network , 610 University Ave , Toronto , ON M5G 2M9 , Canada

## Abstract

While mass spectrometers can detect chemical signatures within milliseconds of data acquisition time, the non-targeted nature of mass spectrometry imaging (MSI) necessitates probing the entire surface of the sample to reveal molecular composition even if the information is only sought from a sample subsection. This leads to long analysis times. Here, we used polarimetry to identify, within a biological tissue, areas of polarimetric heterogeneity indicative of cancer. We were then able to target our MS analysis using polarimetry results to either the cancer region itself or to the cancer margin. A tandem of polarimetry and Desorption Electrospray Ionization Mass Spectrometry Imaging (DESI-MSI) enables fast (10 fold compared to non-targeted imaging), and accurate pathology assessment (cancer typification in less than 2 minutes compared to 30 minutes for histopathology) of *ex vivo* tissue slices, without additional sample preparation. This workflow reduces the overall analysis time of MSI as a research tool.

## Abstract 2

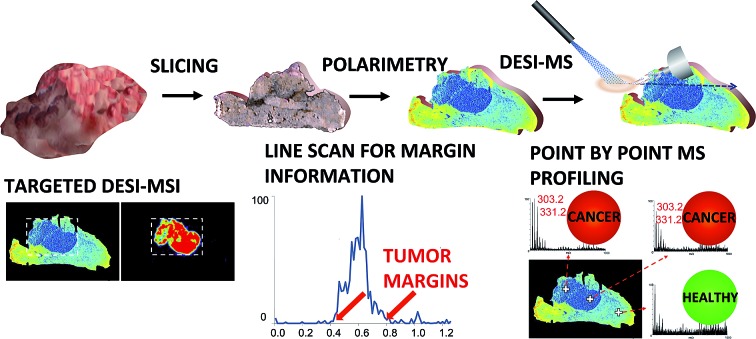
Targeted and localized mass spectrometry imaging allows faster characterization of cancer compared to conventional methods.

## Introduction

The goal of cancer surgery is to remove the entire tumor while sparing as much of the surrounding healthy tissue as possible. In surgical oncology, tumor regrowth due to incomplete resection is a common occurrence in a variety of clinical sites (*e.g.*, breast, liver, skin). To further improve cancer survival and to ensure that the entire tumor is removed in the first (and thus the only) surgery, there is a clinical need for a high-resolution sensitive imaging platform that can discern malignant tumors from normal tissues. Tumors often exist where “wide margin resections” are not possible without creating profound disability in the patient. This further reinforces the need for a high-resolution tumor margin detection platform capable of rapidly detecting even the smallest infiltrating tumors.

A variety of tumor margin estimation approaches are currently under active development, including touch frozen section analysis,^1^ specimen radiography,^2^,^3^ magnetic resonance imaging,^4^ Raman spectroscopy,^5^ radioguided occult lesion localization,^6^ near-IR fluorescence spectroscopy,^7^ Optical Coherence Tomography (OCT)^8^–^12^ and high-frequency ultrasound.^13^ For various reasons, including convenience, availability, sensitivity, information content, operating room workflow compatibility, status-quo and so forth, none of these new methodologies have achieved wide clinical penetration. Thus, intraoperative histology based on microscopy still remains the most accepted approach in routine clinical workflows to determine tumor margins. But this ‘gold standard’ histopathology method is not without its own problems – for example, the process can take up to 30 min while patients await histopathology results under general anaesthesia, and false negatives requiring revision surgery are common (*e.g.* 20% in breast resection^14^). New techniques that offer accelerated delivery of pathology results are thus highly desirable if they can reduce the time and cost associated with tumor resections, without sacrificing (and perhaps even improving) the accuracy of current histopathology assessments.

Mass spectrometry (MS) is a highly sensitive analytic technique that can provide a chemical fingerprint of biological tissues. MS reveals the molecular constituents of tissue on the basis of mass-to-charge (*m*/*z*) ratios in a highly multiplexed manner.^15^ This sensitive technique, with a detection limit on the order of femtomoles, is able to detect hundreds of different molecules in a single measurement.^16^ MS can provide characteristic chemical profiles of a tissue based on its lipid, metabolite or protein composition. Further, MS can be utilized in imaging mode to spatially map the chemical composition of tissues. Mass Spectrometry Imaging (MSI) combines the multiplexed *m*/*z* measurement capability of MS with a surface sampling process to deliver a chemical content map of the target material in a spatially resolved manner. The sensitivity of MS to changes in tissue chemistry makes this technique complementary to other imaging modalities.

Desorption Electrospray Ionization (DESI)^17^ is a recently developed technique in which a spray of charged microdroplets induces desorption and ionization of analytes directly from the surface of an *ex vivo* tissue slice with no other sample preparation. Under ambient conditions, DESI-MS allows identification of tumor sites within healthy tissues on the basis of MS lipid profiles known to be characteristic of cancer.^18^–^24^ More interestingly, DESI-MS also allows further tumor type classification and tumor subclass grading on the basis of unique MS lipid profiles characteristic of each tumor type or subclass.^18^,^20^,^22^,^25^–^27^ For example, it is possible to distinguish between different classes of brain tumors or various subclasses of meningiomas using MS lipid profiles.^22^,^23^,^28^ This has opened up the prospect of intraoperative molecular imaging to identify tumor sites and reveal tumor margins.^18^,^27^ Currently, cross-validation with conventional pathology methods such as histologic staining or immunostaining followed by microscopy is needed to interpret DESI-MS images.^18^,^23^,^29^,^30^ The strong predictive power of MS lipid profiling, in conjunction with robust cancer lipid libraries, make it a leading candidate to become an alternative to histologic staining methods for pathology assessments in the near future. However, current strategies for intraoperative MS data collection from *ex vivo* tissues lead to long analysis times.^18^,^23^,^24^

Note that modern mass spectrometers are capable of delivering robust spectra containing cancer profiles within milliseconds of acquisition time. This has the potential for faster characterization of cancer from *ex vivo* tissue slices than that offered by intraoperative histopathology. To understand the bottleneck that has prevented molecular pathology with MS from competing with intraoperative histology, the workflow for tissue preparation and data collection must be reviewed. For histology, once a slice of tissue is prepared and mounted on a glass slide, conventional pathologic evaluations using Hematoxylin and Eosin (H&E) staining and microscopy may take as little as 15–30 min. Likewise, DESI-MSI is also performed on tissue slices mounted on a glass slide (typically ∼15–20 μm thickness compared to 3–5 μm in H&E). DESI-MSI does not require further processing of the tissue. However, without staining or information from other imaging modalities to guide the DESI solvent spray to areas that are suspected to be cancerous, DESI-MSI often requires imaging the entire excised sample containing healthy and diseased tissues.^18^,^27^ Data acquisition can take anywhere between 30 to 90 min for tissue slices with surface areas of 1 to 2 cm^2^, at ∼150 μm resolution. Therefore, despite the fact that a MS scan in the range of milliseconds is capable of resolving cancer molecular signatures, the need to serially repeat this across the entire tissue specimen area currently makes DESI-MSI considerably slower than intraoperative histology in delineating cancer margins and providing pathology assessment. The increased time compared to intraoperative histology is a major drawback to the clinical adoption of DESI-MSI.

Different approaches have been investigated to reduce MS imaging times. A multimodality imaging approach using optical microscopy and matrix assisted laser desorption ionization mass spectrometry (MALDI-MS) has been introduced by Caprioli and coworkers.^31^ This approach is aimed at obtaining computerized high-resolution molecular images from combining predicted and measured *m*/*z* values from MS, scaled by the higher resolution optical images (H&E); this avoids impractically long MALDI-MSI measurement times of the entire tissue specimen. Previously, through co-registering the MS and microscopy images, molecular MS profiles of tumour microenvironment have been reported.^32^ This pairing, completed after the acquisition of MSI data, facilitates the interpretation of results. However, it provides no means to accelerate a targeted acquisition of MS data from tissue specific segments to save time. Previous studies have reported the pairing of MSI with other imaging modalities such as optical microscopy and Atomic Force Microscopy (AFM) to target molecular mapping to defined points on the surface of a sample.^33^,^34^ Furthermore, MS data were recently reported as stereotactic points onto a 3D MRI image to visualize different grades, and tumor concentrations on a 3D plot of tumor inside the brain.^23^,^35^

In this work we use polarized light imaging to grossly distinguish areas of cancer from surrounding healthy tissue, and utilize this information to guide detailed interrogation of the suspected cancer margins by the more sensitive and specific DESI-MSI, capable of revealing not only tumor margins but also cancer types. This can improve efficiency by an order of magnitude or better. Specifically, we use measured alterations in light polarization to infer tissue biophysical properties, including its local depolarization rates that are sensitive to tissue pathological transformations. These transformations include changes in refractive index heterogeneities stemming from differences in scattering properties of normal *versus* disease cells and associated changes in connective tissue. The so-called Mueller matrix polarimetry imaging provides depolarization maps of tissues with the following advantages: (1) fast measurement times (on the order of tens of seconds); (2) ability to assess large regions of tissues (several square mm to cm); (3) relatively simple and affordable instrumentation; (4) robust low-noise measurements (owing to our recently developed no-moving-parts Mueller matrix imager);^36^ (5) rich and unique information content (above and beyond depolarization metrics that relate to tissue scattering and its spatial heterogeneity, one can derive anisotropy magnitude and orientation that relate to tissue asymmetry/anisotropy); (6) endogenous sources of contrast (no dyes, labels, or other exogenous contrast media are needed).

We have previously investigated Mueller matrix polarimetry for a number of biomedical applications: visualization of myocardial disorganization (loss of anisotropy) following infarction, as well as its regeneration in response to stem cell therapy;^37^,^38^ optical rotation measurements for determining glucose levels in scattering media,^38^,^39^ with potential for non-invasive blood glucose monitoring; detection of morphological changes in the bladder wall due to outlet obstruction disorder.^40^ We have also been active in the technological and theoretical advancement of polarimetry. For example, we recently proposed a no-moving-parts, full-field Mueller matrix polarimeter based on photoelastic modulators,^36^ have investigated the biological validity of different Mueller matrix decomposition methods,^41^ and have developed Monte Carlo simulations for the forward modelling of polarized light transport through turbid media.^42^

Mueller matrix polarimetry imaging is used here to grossly distinguish cancer regions from the background of healthy tissues, in order to guide targeted acquisition of DESI-MS profiling and DESI-MS imaging. In this way, we are able to target the DESI spray on areas of interest such as the tumor region and the border between cancer and healthy tissue for rapid discrimination of cancer margins and cancer type identification. With this targeted DESI-MS approach we are able to detect the presence (or absence) of breast cancer by measuring specific lipid profiles in 1.7 s of data acquisition (two scans of ∼870 millisecond). This targeted profiling approach also allowed mapping positive margins on both sides of a tumor ∼5 mm wide in slightly over 30 seconds of MS acquisition which is an order of magnitude faster than histopathology methods delivering the same information. Polarimetry offers guidance in the absence of any staining and is applicable to the ‘same’ tissue slice being analyzed with MSI. This is significantly different from the image fusion approach described above^31^ that uses an H&E stained slice, consecutive to the one being analyzed by MALDI-MSI to provide guidance.

## Materials and methods

### Animal study

All animal studies were conducted in accordance with institutional guidelines and approved by the animal ethics and use committee (Animal Use Protocol at the University Health Network, Toronto, Canada). Two female Severe Combined ImmunoDeficient (SCID) mice (Harlan) were inoculated with 40 μL of 4 × 10^6^ human MDA-MB-231-LUC breast cancer cells in their quadriceps muscles and housed for 3–4 weeks to allow tumor growth up to 5–7 mm in diameter (determined by calliper measurements). In addition to its immense clinical importance and challenges, breast cancer was an appropriate disease to study because DESI-MS profiles for endogenous, cancer specific lipids have been identified in a number of independent breast cancer studies.^26^,^27^,^43^ In order to create clear boundaries and mimic a breast tumor infiltrating the pectoralis muscles, we inoculated mice with cancer cells in the quadriceps muscle (as opposed to mammary tissue). This allowed wide margin resections with clear definition of the cancer boundaries and easy access to muscle sites for the inoculation.

### *In vivo* imaging

Magnetic Resonance Imaging (MRI) was performed on the day of tumor resection on a 1T – M3 MR system (Aspect Imaging) with a mouse body coil 50 × 30 mm in size. Fast Spin Echo imaging was performed with the following parameters: TE/TR = 54.9 ms/4500 ms, ETL = 16, flip angle = 90°, FOV = 40 × 60 mm, matrix size = 96 × 150, 8 averages and a final pixel size of 0.4 mm.

Bioluminescence Imaging (BLI) was performed one day before tumor resection, on a Xenogen IVIS – 100 Imaging System (Perkin-Elmer). 100 μL of 25 mg mL^–1^d-luciferin potassium salt solution (Perkin-Elmer) was intraperitoneally administered to each mouse and images were acquired 10 minutes post-injection.

### Tissue sample preparation

Mice were sacrificed with an overdose of isoflurane and subjected to surgical removal of the tumors with a wide 2–3 mm margin containing muscle tissue. Extracted tissues were subsequently frozen on liquid N_2_ vapour and stored at –80 °C. Flash frozen tissue was very carefully mounted onto a metal specimen holder with a small amount of Tissue-Tek OCT compound (Sakura Finetek USA Inc), to prevent OCT material from reaching the area being sectioned. Using a CM1950 cryostat (Leica) serial sections with thicknesses of 20 μm, 5 μm and 20–50 μm, for DESI-MS imaging, histological analysis and polarimetry, respectively, were sectioned and mounted onto Superfrost Plus slides. Slides were stored at –80 °C until analysed.

### Laboratory histology analysis

For histological analysis, Hematoxylin & Eosin (H&E) staining was performed as followed: sections were thawed at room temperature for 5 minutes, fixed in 2% paraformaldehyde for 15 minutes and subsequently washed in running tap water for 5 minutes. Tissue sections were then immersed in Harris Hematoxylin (Leica Biosystems) for 3 minutes, washed in warm running tap water for a further 3 minutes before differentiating in 1% acid alcohol. Sections were washed in warm running tap water for 3 minutes prior to immersing in Eosin (Leica Biosystems) for 40 seconds. Sections were washed briefly in water (10 dips) before dehydrating through a series of alcohol solutions from 70% to 100%, cleared through 4 changes of xylene and finally cover slipped using Permount mounting media. Digital images were captured using a TissueScope 4000 slide scanner (Huron Technologies).

### Polarimetry

Polarimetry measurements were made on a homemade polarimetry system operating in transmission geometry. Incident polarization states were produced by passing laser light (635 nm, Thorlabs) through a polarization state generator (PSG), consisting of a linear polarizer and removable quarter wave plate. After interacting with breast cancer samples (20–50 μm slices mounted on glass slides), the polarization state of the emerging light was analyzed under different configurations of the polarization state analyzer (PSA, removable quarter wave plate followed by linear polarizer). The image intensities were then recorded using a CCD camera (CoolSnap K4, Photometrics). Lenses were placed before the sample to generate an appropriate spot size and after the sample to collect and focus the emerging light onto the CCD. Four input polarization states were used: horizontal, vertical, +45° and right circular. Emerging light for each input polarization was analyzed under six different output polarization states: horizontal, vertical, +45°, –45°, right circular and left circular. Hence, for each sample, 24 images were recorded using different PSA/PSG combinations, which were then used to calculate the sample Mueller matrix as previously described.^44^ To extract the polarization parameter of depolarization, Lu–Chipman Mueller matrix decomposition^45^ was used.

### DESI-MS and DESI-MS imaging experiments

All MS experiments were performed using a Thermo Fisher Scientific LTQ mass spectrometer (San Jose, CA, USA). The glass slides containing 20 μm consecutive slices were mounted on a lab-built 2D moving stage (described elsewhere^17^), and subjected to DESI-MS imaging. DESI-MS imaging was carried out in the negative ion mode over the mass range *m*/*z* 260 to 1000. A 1 : 1 mixture of acetonitrile and dimethylformamide (both HPLC-MS grade, Sigma-Aldrich, Oakville, ON, Canada) was used as the charged spray solvent and delivered at a flow rate of 1.5 μL min^–1^. The sprayer-to-surface distance was 1.0 mm, the sprayer to inlet distance was 6–8 mm, an incident spray was set at 52°, and a collection angle of 10° was used. Source parameters were 5 kV capillary voltage, 275 °C capillary temperature, and nitrogen spray at 120 psi. In order to acquire DESI-MS images, tissues were raster-scanned using the laboratory built moving stage described above in horizontal rows separated by 150 μm vertical steps until the entire sample was imaged. The lines were scanned at a constant velocity in the range of 172 to 203 μm s^–1^ and the scan time varied from 0.76 to 0.87 s. The software platform ImageCreator version 3.0 was used to convert the Xcalibur 2.0 mass spectra files (.raw) into a format compatible with BioMap (freeware, ; http://www.maldi-msi.org/), which was used to process the mass spectral data and to generate 2D spatially resolved ion images. The assignment of lipid biomarkers seen in the negative ion mode of the tumor samples was made by DESI-MS/MS, corroborated with published breast cancer MS profiles.^26^,^27^,^43^

## Results and discussion

To evaluate the utility of wide-field tissue polarimetry in guiding DESI-MS we used human breast cancer cells grown into tumors inside quadriceps muscles of mice. *In vivo* Magnetic Resonance (MR) images and bioluminescence images were acquired prior to tumor resection to evaluate size and location (Fig. S1†). Our model mimics posterior breast cancer tumors that are often attached to, and infiltrate the pectoralis major muscle. These tumors are difficult to visualize using mammography.^46^

**Fig. 1:**
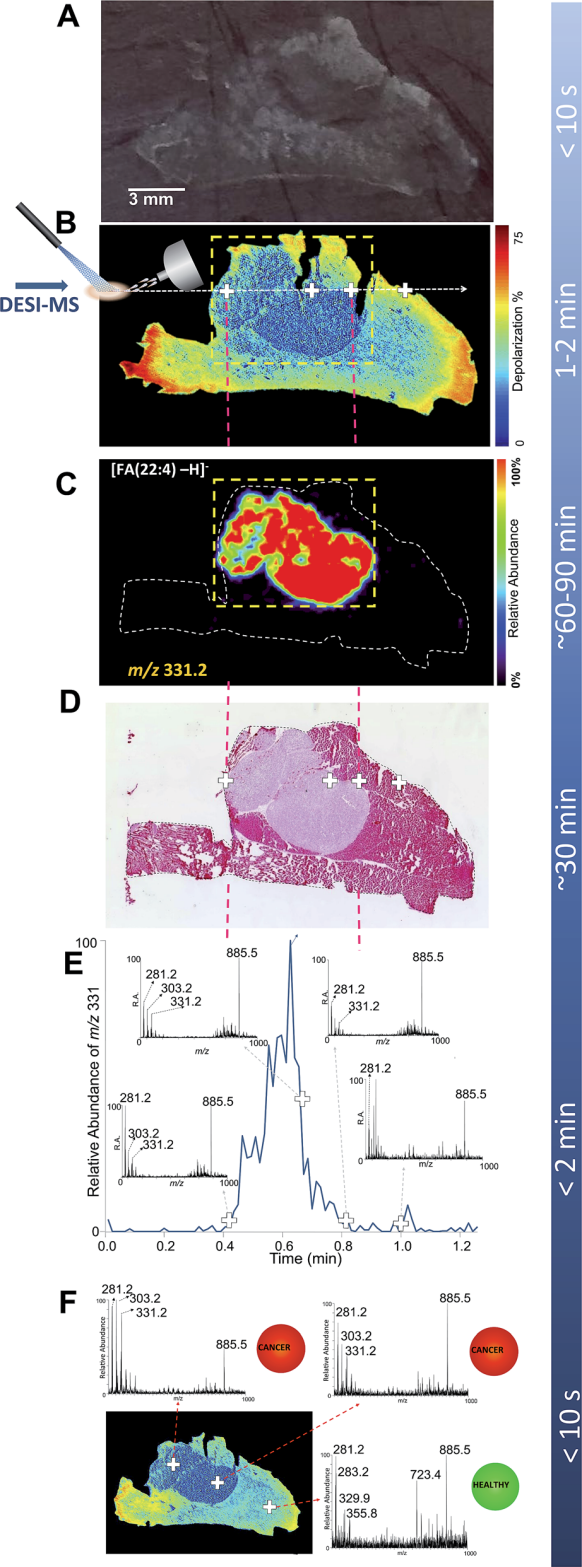
DESI-MSI and Mueller matrix polarimetry imaging of an infiltrating breast cancer tumor. (A) Optical image of a 20 micron thick tissue slice comprising a breast cancer tumor that infiltrates the adjacent muscle tissue. (B) Mueller matrix polarimetry image of the same tissue as in A. (C) DESI-MSI of a breast cancer marker of *m*/*z* 331.2 corresponding to andrenic acid [FA(22:4)-H]^–^. Here, a focused DESI-MSI area revealed by polarimetry to be cancerous (box, dashed yellow line) can be imaged. (D) The H&E image of a consecutive tissue slice highlighting the cancer region. (E) The extracted ion chromatogram for one breast cancer marker, andrenic acid [FA(22:4)-H]^–^ of *m*/*z* 331.2, collected from a line scan corresponding to the dashed white line in the polarimetry image (panel B). The insets to panel C show MS spectra averaged over two instrument scans from select points at the margins, in the middle of the tumor tissue, as well as in the muscle, as highlighted with crosses on the polarimetry image. (F) DESI-MS point scans to reveal presence of cancer at given points on the tissue sample. This process could be automated with tissue classification information being displayed to the clinician/pathologist in a straight-forward, easy-to-interpret manner (*e.g.*, color coded red for cancer and green for healthy tissues as reported previously^52^).

In a typical white light optical image of the tissue slice (Fig. 1A), as presented to the mass spectrometer operator, the breast cancer region infiltrating healthy muscle is not readily visible to the naked eye. With no visual cues to guide the placement of DESI solvent spray on the tumor region and the boundary between healthy and cancerous tissue, the MS operator would have to image the entire tissue slice.

In contrast with white light imaging, Fig. 1B illustrates that wide-field polarimetry of the same tissue (in this case a consecutive slice, see Fig. S2†) quickly reveals areas of heterogeneity based on different depolarizations induced by the healthy and cancer regions. We observed that breast cancer was less depolarizing compared to the surrounding muscle tissue. Similar findings have been previously reported for colon^47^ and cervical cancer.^48^ For example, Antonelli *et al.* showed that early stage colon cancer was less depolarizing than surrounding healthy tissues.^47^ Depolarization due to multiple light scattering is a consequence of the turbid heterogeneous nature of tissue. The amount of depolarization is influenced by tissue parameters such as scatterer (cells, nuclei, connective tissue fibers, *etc.*) sizes, shapes, and densities, all causing complex spatial variations in optical refractive index patterns. In cancer, tissue architecture is significantly altered^49^ (*i.e.* increased nuclear density and size, stromal alterations) resulting in different depolarization patterns than healthy tissue. Here, the observed depolarization contrast is likely also influenced by anisotropy (alignment) of the muscle background. It has been suggested that anisotropic tissues (such as fibrous muscle) exhibit increased depolarization due to spatially inhomogeneous microdomains of varying linear retardance/birefringence.^50^ As polarized light passes through these spatially varying regions, it undergoes additional randomization and hence depolarization increases.

To validate that polarimetry can indeed reveal breast cancer regions, a DESI-MS image of the entire tissue slice was performed (Fig. 1C). Here we present a map of the ion of *m*/*z* 331.2, a prominent lipid marker of breast cancer^26^,^27^,^43^ identified as andrenic acid [FA(22:4)-H]^–^. The cancerous region of increased andrenic acid indeed corresponds to the area suggested by polarimetric images to contain tissue material with an altered morphology. Comparison with the H&E image (Fig. 1D) confirms the results; areas shown through polarimetry to be less depolarizing are indeed cancerous. Fig. 1C demonstrates how polarimetry can be used to select a smaller region (shown by the dashed-line box) containing only the tumor for selective analysis with DESI-MSI, eliminating the need to image the entire tissue slice.

The insets in Fig. 1E show MS spectra obtained from four select points (marked with crosses on the polarimetry image, Fig. 1B) of the tissue: two points at the tumor margins, a typical point inside the tumor, and a point in the healthy muscle tissue. Each spectrum was averaged over two scans, with a total acquisition time of 1.7 s for each spectrum. Highlighted in the spectra are major lipid markers characteristic of breast cancer: *m*/*z* 281.2 corresponding to oleic acid [FA(18:1)-H]^–^, *m*/*z* 303.2 for arachidonic acid [FA(20:4)-H]^–^, *m*/*z* 331.2 for andrenic acid [FA(22:4)-H]^–^ as well as the phospholipid species of *m*/*z* 885.5 identified as [PI(38:4)-H]^–^.^26^,^27^,^43^ These observations are in agreement with findings presented in Calligaris *et al.*,^27^ suggesting that about 85% of breast cancer samples have a significant increase in ion abundance in the low-mass region (*i.e.*, below *m*/*z* 700) such as ions of *m*/*z* 303.2 and 331.2. The ions of high mass range, for example *m*/*z* 885.5, exist in both tumor and normal specimens. All of these markers were confirmed by DESI-MS/MS (Fig. S3–S6†). These markers have been observed with DESI-MSI in studies of intraoperative tissue biopsies^43^ and in a patient cohort undergoing double mastectomies.^27^ The fact that the human MDA-MB-231 breast cancer cell line used in this study presents these same lipid markers supports the choice of our cancer mouse model, *via* its potential clinical relevance. As expected, the mass spectrum from the muscle region did not reveal characteristic breast cancer markers.

In the absence of feedback from other imaging modalities, mapping cancer borders with DESI-MS is achieved by analyzing the distribution of cancer markers across the entire tissue sample (as seen in Fig. 1C). In the interest of reduced analysis time, it has been recently suggested that a line scan (*i.e.* MS profile along a line through the tumor) may be sufficient for understanding 1-dimensional cancer margins in an excised sample intraoperatively.^27^ This is achieved through monitoring the rise and fall of cancer marker ion intensity in the extracted ion chromatogram. However, for rapid assessment of tumor margins and characterization of cancer type, the line scan should target the cancer region and the border between cancer and healthy tissue. Therefore, to effectively target the DESI solvent spray, an understanding of the approximate location of the cancer region/healthy tissue transition is desirable. Fig. 1E illustrates a targeted DESI-MS ion chromatogram for one of these cancer markers, andrenic acid [FA(22:4)-H]^–^ of *m*/*z* 331.2. Here, the placement of DESI spray was guided by polarimetry to a line across the area suspected to be cancerous. This ion chromatogram corresponds to a line scan across the sample as indicated in Fig. 1B (dashed white line). The total scan time for this line was 72 seconds. By strategically placing the DESI solvent spray to the area revealed by polarimetry to be likely cancerous, we were able to determine tumor margins from the rise in the intensity of the andrenic acid ion in approximately one minute of data acquisition. There is a strong correspondence between the rise along the MS scan line of the andrenic acid ion intensity and the boundary of the tumor as revealed through H&E staining (Fig. 1D).

Typical procedure times for various experiments are also listed in Fig. 1. A combined polarimetry guided DESI-MS line scan is capable of elucidating 1-dimensional cancer border in less than 3 minutes (2 minutes for polarimetry and slightly over 30 seconds for a continuous DESI-MS line scan crossing the tumor boundaries). This constitutes a 10-fold acceleration in margin assessment compared to H&E delivering boundary information. It must be emphasized that to image the *entire* tumor boundary (2D margin information), a tandem of polarimetry and DESI-MSI can be used to reduce the effective area to be imaged to one that immediately surrounds the suspected cancer region (boxed area in Fig. 1C). The total analysis time for the highlighted section revealing the entire cancer area (0.70 cm × 0.55 cm) is close to 12 minutes, which is still 2.5 times faster than H&E methods. With our guided imaging approach, tumors 1.5 cm × 1.5 cm in extent can be entirely mapped to reveal 2D cancer boundaries in 30 minutes of MSI acquisition. With more rapid MS imaging technologies such as high speed MALDI-MS imaging being developed,^51^ polarimetry guided MS imaging of biological tissues may become even faster than we have demonstrated here. In addition, a MALDI-MS imaging approach may further improve spatial resolution.

Finally, Fig. 1F illustrates how a point-by-point profiling could be achieved using guided DESI-MS to rapidly typify the cancerous tissues in select points within the regions suspected to be pathologic by polarimetry. Here we show three representative spectra from various points (marked with crosses) within both the cancer region as well as from within the healthy muscle tissue, with prominent cancer markers *m*/*z* 303.2 and 331.2 highlighted. Additionally, the presence or absence of cancer could be translated into easy-to-interpret color map indicators for rapid feedback to clinicians and pathologists, as recently suggested.^52^

**Fig. 2:**
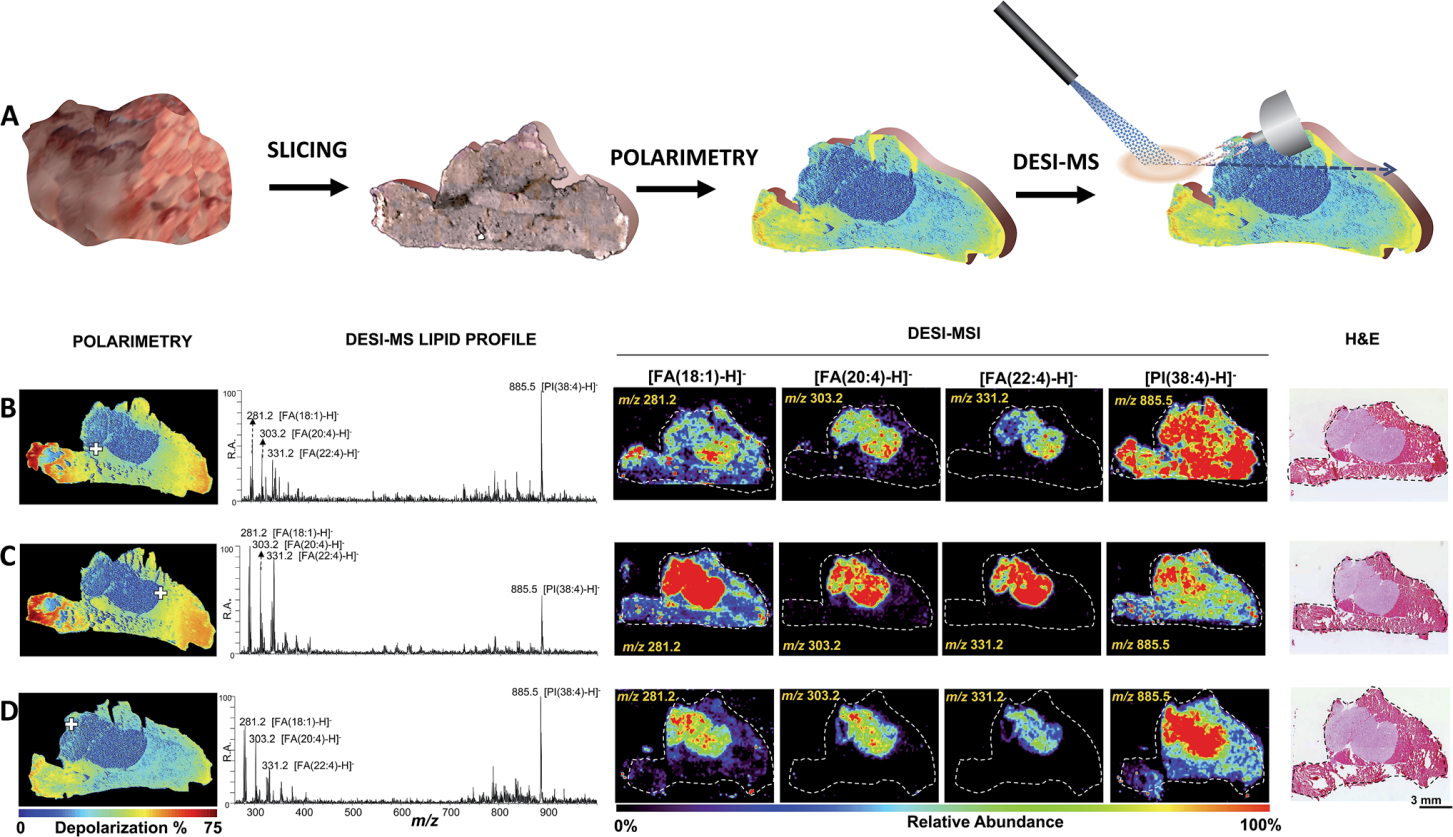
Polarimetry guided DESI-MS analysis of breast cancer. (A) Polarimetry/DESI-MS work flow. The tissue is sliced and mounted on a glass slide. The section is then imaged *via* wide-field polarimetry revealing suspected cancer regions from differential depolarization. The same slide is then subjected to DESI-MS or DESI-MSI analysis. (B–D) Analysis of three tissue slices by tandem of Mueller matrix polarimetry and DESI-MS. From left to right: polarimetry depolarization images, DESI-MS lipid profiles collected at a typical point in the tumor margin (highlighted with a cross over the polarimetry image), DESI-MSI of breast cancer marker ions [FA(18:1)-H]^–^ of *m*/*z* 281.2, [FA(20:4)-H]^–^ of *m*/*z* 303.2, [FA(22:4)-H]^–^ of *m*/*z* 331.2 and [PI(38:4)-H]^–^ of *m*/*z* 885.5, as well as H&E images are shown. The position of the DESI spray for the strategic collection of MS spectra was guided by polarimetry. The results shown are consistent with those from an independent mouse presented in Fig. S7.†

To evaluate the reproducibility of this approach, Fig. 2 shows the correspondence between DESI-MSI and Mueller matrix polarimetry images of three different slices of breast cancer tumor infiltrating muscle tissue. A consecutive slice was subjected to conventional H&E analysis for corroboration, as per the tissue preparation work-flow described in Fig. S1.† In all three cases DESI-MSI revealed elevated relative abundances of the breast cancer markers [FA(20:4)-H]^–^ of *m*/*z* 303.2, [FA(22:4)-H]^–^ of *m*/*z* 331.2 and [FA(18:1)-H]^–^ of *m*/*z* 281.2 in areas identified through polarimetry and H&E to be cancerous. Fig. 2 also shows MS spectra collected at positive tumor margins (indicated with a cross) containing all of the breast cancer markers described above.

We further evaluated the robustness of polarimetry guided DESI-MSI using another breast cancer tumor grown in a second SCID mouse. Fig. S7† illustrates the results, once again highlighting correspondence between DESI-MSI, polarimetry and H&E in cancer identification. That is, regions revealed by histology to be cancerous correspond to regions of lower depolarization (measured with polarimetry) and elevated relative abundances of breast cancer markers (measured with DESI-MSI).

In summary, multiple ways in which the tandem of polarimetry and DESI-MS profiling could potentially be implemented in a clinical setting (workflow) are illustrated in Fig. 1. In all cases a tissue section 20 μm in thickness is prepared from an excised sample, and is mounted on a glass slide. Polarimetry is then performed (1–2 min) to grossly reveal cancer regions. The same slice is then subjected to guided DESI-MS profiling. This profiling could be done in a number of ways: (1) accelerated targeted 2D DESI-MSI (Fig. 1C); (2) accelerated 1D margin assessment (Fig. 1E); (3) point-by-point DESI-MS profiling (Fig. 1F) to reveal cancer type. Table S1† summarizes tissue preparation requirements as well as analysis times for all imaging modalities used in this study.

Polarimetric information (*e.g.*, depolarization images) is indicative of the heterogeneous nature of the underlying tissue microstructure, and may thus lack chemical specificity for cancer detection (as afforded by DESI-MSI). This could lead to incorrect “targeting” for the tandem MS analysis, a potentially important limitation of the proposed methodology that needs to be rigorously evaluated. One approach to improve the polarimetric specificity (and thus reduce potential false positives and false negatives) will be to include other Mueller matrix metrics available for differential tissue analysis (*e.g.*, birefringence images). Further, the tandem combination proposed here will enable the DESI-MSI to conclusively confirm the absence/presence of tumor in the targeted image region, thus “correcting” the polarimetric false positives. These issues are complex, and will be examined and refined in future studies. Owing to its superior sensitivity, DESI-MSI may distinguish tumor infiltration on the basis of MS profiles.^18^ However, low concentration of cancer cells present in such areas (few isolated cancers cells surrounded by normal cells) could still present a challenge in cancer detection by DESI-MSI; the technique's ultimate detection limit is yet to be determined.

## Conclusions

In this work we demonstrate the utility of combined polarimetry and DESI-MSI for accelerated identification of tumor boundaries. Polarimetry images are made available considerably faster than H&E images proposed for guiding MSI. Therefore, a multi-modality combination of polarimetry and MSI appears capable of accelerating the acquisition of MSI data. Through polarimetry and DESI-MS fusion, the different strengths of these two techniques are accessed. Using polarimetry-targeted MS analysis, it may become feasible to perform intraoperative molecular pathology evaluations more rapidly than currently possible with conventional non-targeted MSI methods, while retaining the relevant information content afforded by histology. This may have significant implications for the current pathology assessment and work-flow in the operating room. Coupling between polarimetry and other MSI technologies such as MALDI-MS is possible and could be pursued. The sensitivity of MS may also make this technology amenable to early diagnosis of cancer where minute quantities of cancer markers detected could indicate pathology. The utility of a polarimetry/MSI tandem for early detection of cancer will be investigated in future work.

## Supplementary Material

Supplementary information
