# Supplementary material for: Wide-field tissue polarimetry allows efficient localized mass spectrometry imaging of biological tissues
Source: Chem Sci. 2015 Dec 15;7(3):2162–9. doi: 10.1039/c5sc03782d (PMC6090527; doi:10.1039/c5sc03782d)
Supplement: Supplementary file 1 [file SC-007-C5SC03782D-s001.pdf]

Electronic Supplementary Material (ESI) for Chemical Science.
This journal is © The Royal Society of Chemistry 2015

Supporting Information

Wide-Field Tissue Polarimetry Allows Efficient Localized Mass Spectrometry Imaging of Biological Tissues

Alessandra Tata[a], Adam Gribble[b], Manuela Ventura[a], Milan Ganguly[c], Emma Bluemke[a,b], Howard J. Ginsberg[a,d,e] , David A. Jaffray[a,b], Demian R. Ifa[f], Alex Vitkin[b,g,h] and Arash Zarrine-Afsar*[,a,b,d,e]

[a]Techna Institute for the Advancement of Technology for Health, University Health Network, Toronto, ON, M5G-1P5, Canada
[b]Department of Medical Biophysics, University of Toronto,101 College Street Suite 15-701, Toronto, ON, M5G 1L7, Canada
[c]STTARR Innovation Centre, Princess Margaret Cancer Centre, 101 College Street, Toronto, ON M5G 1L7
[d]Department of Surgery, University of Toronto, 149 College Street, Toronto, ON, M5T-1P5, Canada
[e]Keenan Research Centre for Biomedical Science, Li Ka Shing Knowledge Institute, St. Michael's Hospital, 30 Bond Street, Toronto, ON, M5B-1W8, Canada
[f]Department of Chemistry, York University, 4700 Keele Street, Toronto, ON, M3J-1P3, Canad
[g]Department of Radiation Oncology, University of Toronto, 610 University Avenue, Toronto, Ontario M5G 2M9, Canada
[h]Division of Biophysics and Bioimaging, Ontario Cancer Institute, University Health Network, 610 University Ave, Toronto, ON M5G 2M9

*Correspondence to arash.zarrine.afsar@utoronto.ca

Table of Contents

Figure S1……………………………………….Page S3
Figure S2……………………………………….Page S4
Figure S3……………………………………….Page S5
Figure S4……………………………………….Page S6
Figure S5……………………………………….Page S7
Figure S6……………………………………….Page S8
Figure S7……………………………………....Page S9
Table S1……………………………………....Page S10

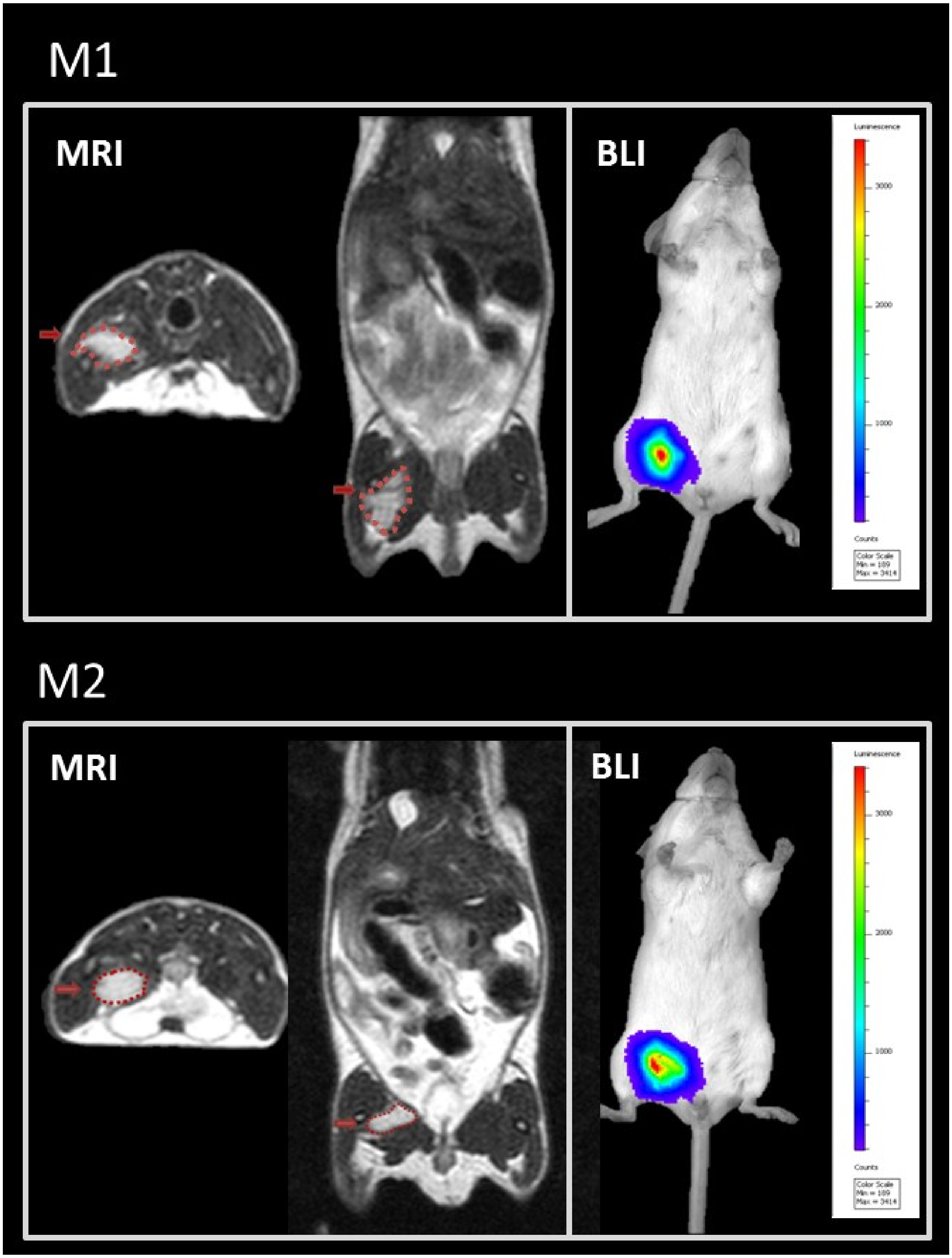

Fig. S1. ***In Vivo*** **imaging of mouse 1 (M1) and mouse 2 (M2).** Axial and coronal 2D-MR slices were used to verify location and morphology of the tumors within the muscle (highlighted by the red arrows and dashed lines), as well as for tumor volume measurements. Bioluminescence Imaging (BLI) was also performed to confirm tumor location and viability.

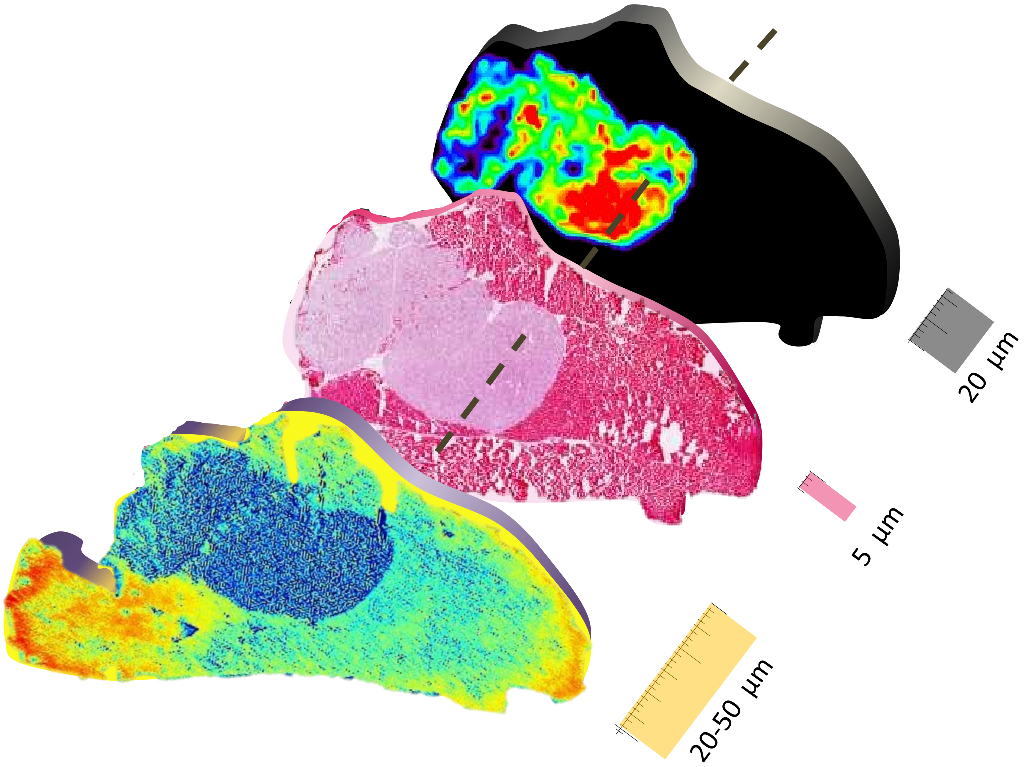

Fig. S2. Tissue Sectioning. Sectioning of serial slices of 20-50 µm, 5µm and 20 µm submitted to wide field tissue polarimetry, H&E staining and DESI-MSI, respectively. In the future, the same slice of 20 µm thickness can be subjected to DESI-MSI after polarimetry (as described in Figure 2).

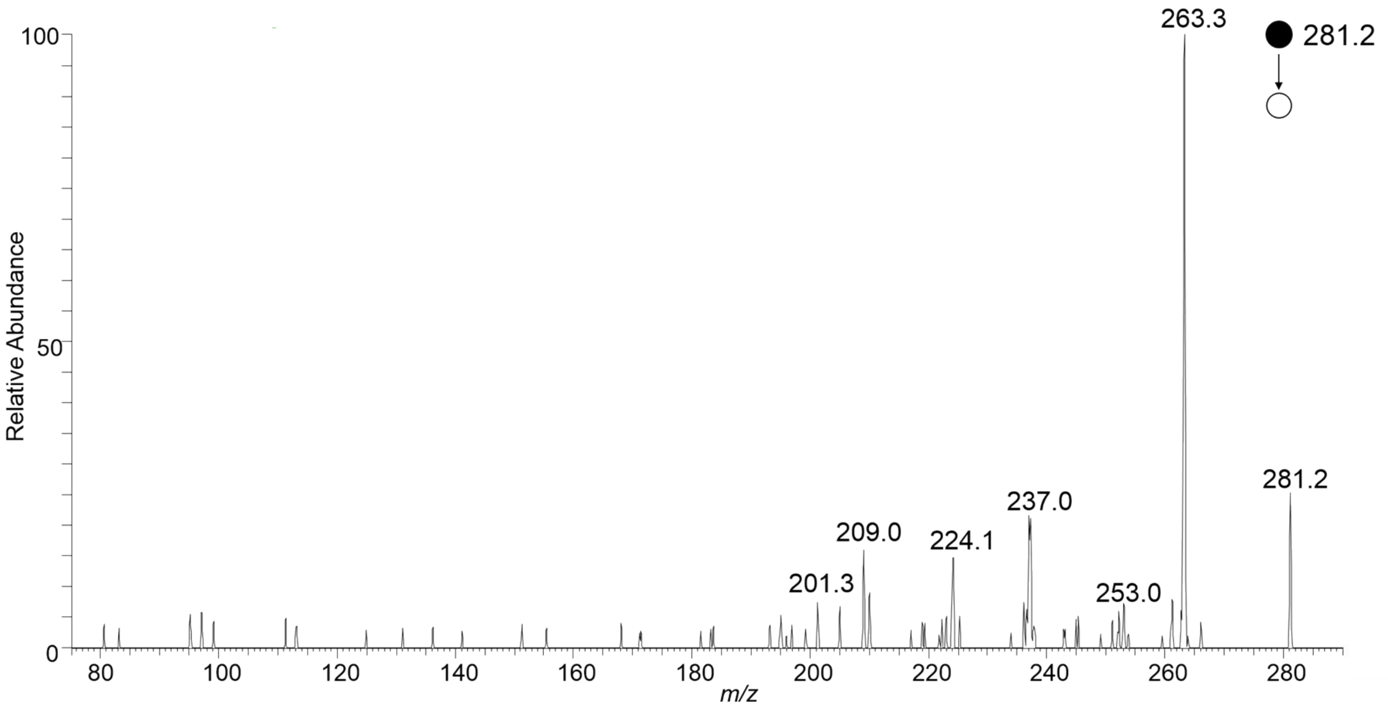

Fig. S3. DESI-MS/MS spectrum of the ion of *m/z* 281.2 identified as oleic acid.

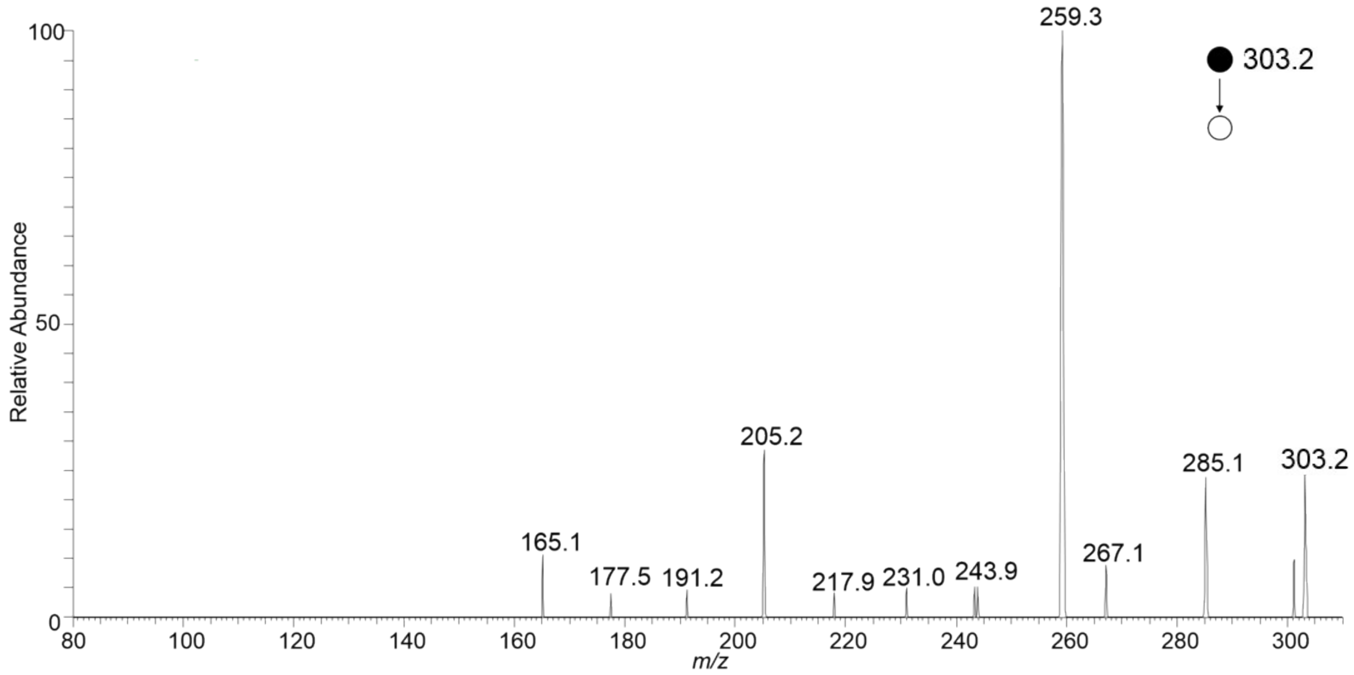

Fig. S4. DESI-MS/MS spectrum of the ion of *m/z* 303.2 identified as arachidonic acid

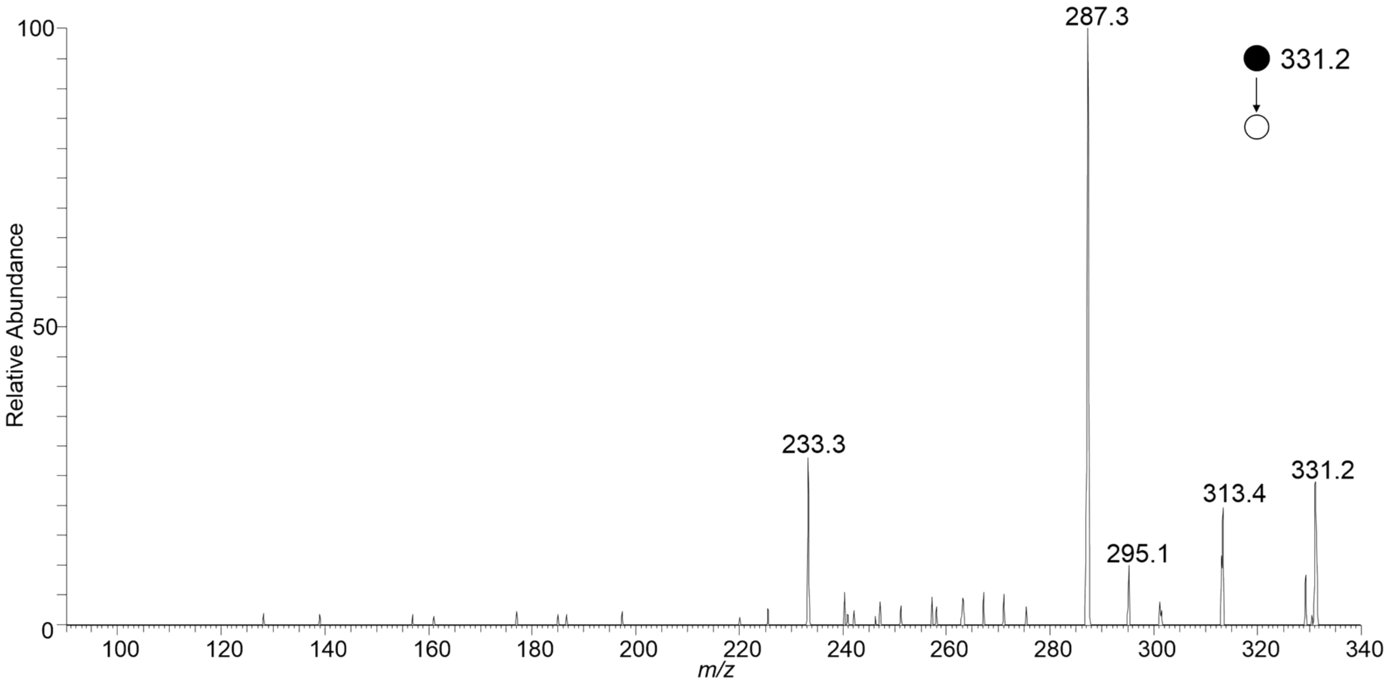

Fig. S5. DESI-MS/MS spectrum of the ion of *m/z* 331.2 identified as andrenic acid.

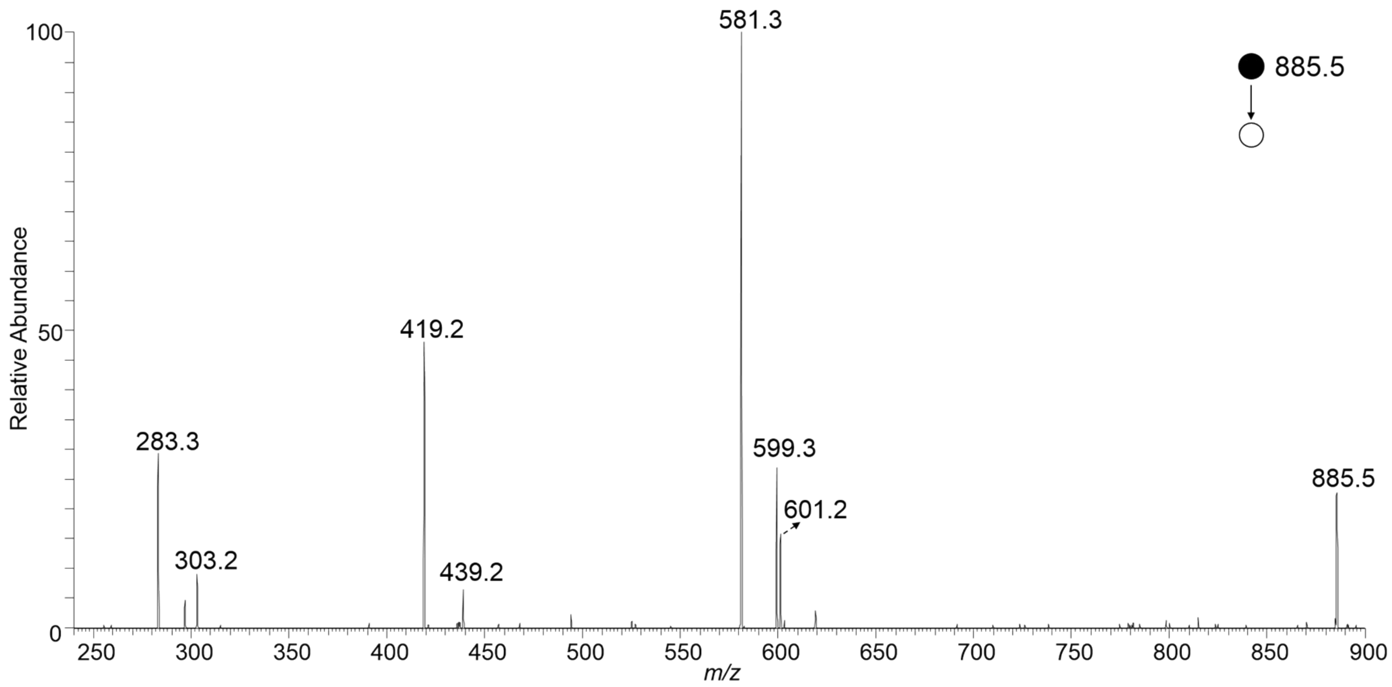

Fig. S6. DESI-MS/MS spectrum of the ion of *m/z*885.5identified as [PI(38:4)-H]$^{-}$

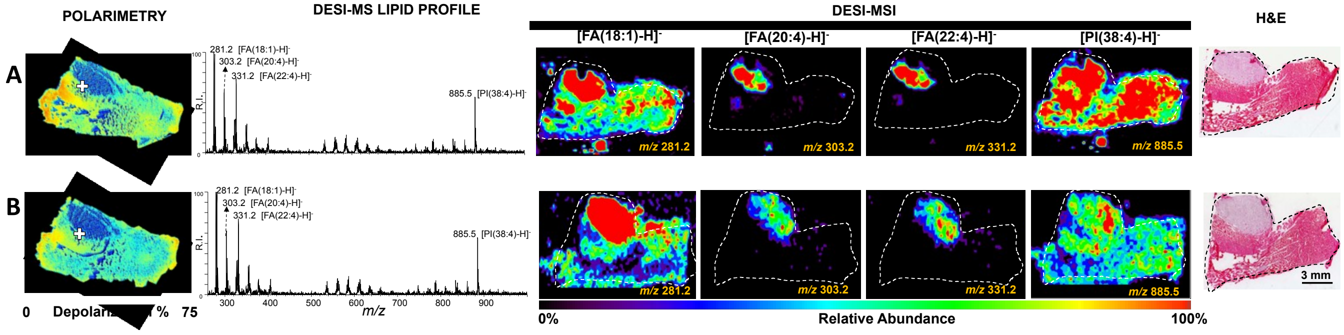

Figure S7. Analysis of two tissue slices (A,B) by tandem of Mueller matrix polarimetry and DESI-MSI: reproducibility check in a different mouse. From left to right: Polarimetry depolarization images, DESI-MS lipid profiles collected at a typical point in the tumor margin (highlighted with a cross over the polarimetry image), DESI-MSI of breast cancer marker ions $[FA(18:1)-H]^-$ of *m/z* 281.2, $[FA(20:4)-H]^-$ of *m/z* 303.2, $[FA(22:4)-H]^-$ of *m/z* 331.2 and $[PI(38:4)-H]^-$ of *m/z* 885.5, as well as H&E images are shown. The position of the DESI spray for the strategic collection of MS spectra was guided by polarimetry. The results shown are consistent with those from an independent mouse presented in Figure 2.

Table S1. Summary of the relevant imaging times, thicknesses and sample preparation used in this study.

Imaging technique	Tissue preparation methods	Tissue thickness (µm)	Time to final result (min)
**WIDE-FIELD POLARIMETRY***	Snap frozen, cryo-sectioned and thaw mounted on a glass slide.	20-50	1-2 (variable field of view, up to several cm^2)
**DESI-MS**		10-50	~30-90 min for a $1cm^2$ area
**H&E STAINING & MICROSCOPY**		4-10	~30 minutes

***** Note that all the polarimetry parameters can be adjusted or improved. The values shown here were selected to comply with the DESI-MS work flow requirements.
